# Interferon Alpha Characterization and Its Comparative Expression in PBM Cells of *Capra hircus* and *Antelope cervicapra* Cultured in the Presence of TLR9 Agonist

**DOI:** 10.4061/2010/573426

**Published:** 2010-06-03

**Authors:** Ramesh Doreswamy, Mohini Saini, Devendra Swarup, Vivek Kumar Singh, Suchitra Upreti, Asit Das, Praveen K. Gupta

**Affiliations:** ^1^Centre for Wildlife, Indian Veterinary Research Institute, Izatnagar, Uttar Pradesh 243 122, India; ^2^Division of Animal Biotechnology, Indian Veterinary Research Institute, Izatnagar, Uttar Pradesh 243 122, India

## Abstract

TLR9 plays pivotal role in innate immune responses through upregulation of costimulatory molecules and induction of proinflammatory cytokines like type I interferons including interferon alpha (IFNA). The present study characterized IFNA cDNA and predicted protein sequences in goat and black buck. Response of the PBM cells to TLR9 agonist CpG ODN C and Phorbol Myristate Acetate (PMA) was evaluated by realtime PCR. IFNA coding sequences were amplified from leukocyte cDNA and cloned in pGEMT-easy vector for nucleotide sequencing. Sequence analysis revealed 570 bp, IFNA ORF encoding 189 amino acids in goat and black buck. Black buck and goat IFNA has 92.1% to 94.7% and 93% to 95.6% similarity at nucleotide level, 86.3% to 89.5% and 70.9% to 91.6% identity at amino acid level with other ruminants, respectively. Nonsynonymous substitutions exceeding synonymous substitutions indicated IFNA evolved through positive selection among ruminants. In spite of lower total leukocyte count, the innate immune cells like monocytes and neutrophils were more in black buck compared to goat. In addition, CpG ODN C-stimulated PBM cells revealed raised IFNA transcript in black buck than goat. These findings indicate sturdy genetically governed immune system in wild antelope black buck compared to domestic ruminant goat.

## 1. Introduction

TLR9 emerged as a robust pathogen recognition receptor of innate immune system in the present decade and is known to be present in the endosomal membrane of the Plasmacytoid dendritic cells [pDCs], macrophages, and B- cells where it functions as a known sensor for bacterial, viral, fungal, and parasitic foreign DNA [1]. pDCs have been proved as powerful natural Interferon alpha (IFNA) secreting cells in mouse, human, and porcine in response to bacterial/viral (unmethylated cytosine-guanine) CpG motifs via TLR9 signaling [2–4]. 

Unmethylated CpG motifs or pathogen derived DNA rich in CpG motifs are known to be the cognate agonists for TLR9. In response to TLR9 signaling, several downstream co-stimulatory molecules and cytokines like Type I interferons (IFN), IFN-*γ*, Interleukins IL1, IL6, IL10, IL12, and IL18, involved in evoking the adaptive immune system optimally against the invading pathogen, are produced. Among the type I IFNs, IFNA (leukocyte) is of prime importance as earliest cytokine activating macrophages and NK cells to collectively bring Th1 response. TLR9 signaling is involved in recognizing and stimulating the adaptive immune system against the invading pathogen like blood protozoan in *Leishmania* major [5], *Trypanosoma cruzi* [6]. Moreover, CpG (oligodeoxynucleotide) ODN is used as immunotherapeutic agents in clinical trials for cancer [7] and infectious diseases like influenza reviewed by [8] and parasitic diseases like Toxoplasmosis [9].

Adaptive immunity has been intensively studied in animals whereas cellular responses in innate immunity have been poorly understood. Our previous study has shown higher basal expression of innate immune marker like TLR2 and TLR3 in immune cells of wild ruminant nilgai as compared to domestic counterpart cattle and buffalo [10, 11]. The mechanisms and roles of innate immune responses could be distinct between genetically diverse wild versus domestic animals. Blackbuck (*Antelope cervicapra*), mainly distributed in open plains of India and parts of Pakistan and Nepal, is an endangered species of antelope; included and protected under Schedule I of “The Indian Wildlife Protection Act (WPA, 1972)” and has been declared as near threatened species by IUCN Red Data Book (2009). The present work reports IFNA sequence and the comparison of PBM cell responses following TLR9 signaling, in black buck *(Antelope cervicapra)* and goat* (capra hircus)*.

## 2. Materials and Methods

### 2.1. Isolation of PBM Cells

Blood was collected aseptically by jugular venipuncture using 2.7% EDTA from the healthy Goat (*Capra hircus*) and Black buck (*Antelope cervicapra*) reared at the Deer Park of Centre for Wildlife, IVRI. Hellbrunns mixture (ketamine : xylazine = 1 : 1.25) at 0.25–0.5 mL was used for tranquilization and capture of black buck. Before going for the actual analysis of blood, total leukocyte count (TLC) and differential leukocyte count (DLC) ware performed in order to assess cellular status of their immune system. Then Peripheral blood mononuclear cells (PBM cells) were isolated from whole blood by density gradient centrifugation [12] using Histopaque (Sigma, USA.). Since IFNA is being a leukocytic IFN constitutively expressed in these cells, the cells were harvested in Trizol for total RNA isolation.

### 2.2. Total RNA Isolation and cDNA Synthesis

Total RNA was isolated using Trizol LS reagent (Life Technologies, New York, NY) following the manufacturer's instructions. The 20 *μ*L reaction mixture contained 5 *μ*g of total RNA, 0.5 *μ*g of oligo dT primer (16–18 mer), 40 U of RNasin, 10 mM of dNTP mix, 10 mM of DTT, 5 U of MuMLV reverse transcriptase in 1× reverse transcriptase buffer using (Fermentas, USA). The reaction mix was incubated at 37°C for 1 hour. The cDNA synthesis was confirmed by amplifying the GAPDH amplicon in PCR.

### 2.3. Polymerase Chain Reaction of IFN Alpha

Based on the consensus IFNA mRNA sequences of cattle (E00135.1), horse (M14542.1), domestic cat (NM_001009963.1), sheep (AY802986.1), primate giant panda (DQ392970.1), pig (DQ872656.1), water buffalo (AY323972.1) primers were designed using Oligo 4 software. To amplify the complete full length ORF (570 bp) of IFNA gene in black buck and goat, the primer pairs include IFNA Forward 5′-CAATGGCSCYRSCCTGKTCCTT-3′ and Reverse 5′- CCAGGTGTGTGTCAKTCCTTYCTCCT-3′. Likewise for nested PCR (140 bp) internal primers included Forward 5′-GAAGGCTCAAGCCATCTCTG-3′ and Reverse 5′-GGTCAGTGAGCTGCTGATCC-3′ (*K = T or G, S = C or G). The PCR reaction mixture of final volume 50 *μ*L contained 50 pmol for each forward and reverse primers, 1 *μ*L template cDNA, 200 *μ*M each of dNTP mix, 1.0 mM MgCl2, and 2.5 U proofreading DNA polymerase (MBI Fermentas, USA) in 1× Taq buffer. 

PCR reaction condition follows initial denaturation at 95°C for 5 minutes, followed by 35 cycles of 94°C for 45 seconds, 60°C for 45 seconds; 72°C for 90 seconds, and final extension at 72°C for 10 minutes. The PCR amplicon (598 bp) along with the 100 bp DNA ladder were resolved by 1% agarose gel electrophoresis. PCR amplicons were purified and ligated into pGEMT easy cloning vector (Promega, Madison, WI, USA) following manufacturer's instructions. Recombinant plasmids were characterized by using restriction enzyme EcoRI (MBI Fermentas, USA) for insert release (598 bp). Recombinant plasmids with IFNA gene fragments of both the species were sequenced using M13 Forward primers in Automatic Sequencer by Chromous Biotech Pvt. Ltd, Bangalore.

### 2.4. Sequence Analysis

The sequences of the obtained fragments were aligned using DNA star MegAlign software (Lasergene, USA) and complete full length ORF (570 bp) sequences of Black Buck and Goat were identified. The domain structure, glycoyslation sites, phosphorylation sites, and hairpin loop structure were determined by online software like, PROSITE (http://www.expasy.ch/pro), SMART (http://smart.embl-heidelberg.de/), and SWISS MODEL. Phylogenetic tree based on the evolutionary distances was constructed using Mega 4.1 [13], based on the nucleic acid alignment, the number of synonymous substitution per synonymous site (*dS*) and number of non-synonymous substitution per nonsynonymous sites (*dN*) were estimated, and neutral (*dS* = *dN*), positive (*dN* > *dS*), or purifying (*dN* < *dS*) selections were tested with a codon-based Z test using Nei Gojobori method.

### 2.5. IFN Alpha mRNA Quantification in PBM Cells In Vitro

Blood was collected from goat and black buck and PBM cells were isolated as described above and cultured in RPMI 1640 (Sigma, USA) containing 10% FCS (Sigma, USA) with optimum cell concentration must be of 2 × 10^6^ cells per mL. Cells were treated with mitogen (Phorbol Myristate Acetate) PMA (Sigma USA) 10 ng/mL, TLR9 agonist CpG ODN type C (Invivogen, USA) 10 *μ*g/mL along with unstimulated cells. After 18 hours of incubation at 37°C in a CO_2_ incubator, the cells were harvested and kept in Trizol at −20°C till further processed. Total RNA isolation and cDNA synthesis was performed as described above. For quantifying the IFNA mRNA expression, quantitative real-time PCR assay was performed using nested PCR primers primer pairs for IFNA (internal fragment of 140 bp) and GAPDH (200 bp) genes as described by Menzies and Ingham [14]. Real-time PCR was performed using Brilliant SYBER Green QPCR Mastermix (Stratagene, USA) and Mx3000P Spectrofluoremetric thermalcycler operated by MxPro*™* QPCR software. The experiment was repeated twice and each QPCR reaction was put in triplicate in a total volume of 25 *μ*L each, which contained 1 *μ*L each of forward and reverse primers (10 pmol/*μ*L), 1.0 *μ*L of template cDNA, 9.5 *μ*L nuclease-free PCR grade water, and 12.5 *μ*L of 2X QPCR mastermix. No template control (NTC) was put for each gene quantification for checking the contamination in the reaction components other than the cDNA. The PCR condition was as follows: initial denaturation at 94°C for 3 minutes, followed by 40 cycles consisting denaturation at 94°C for 30 seconds, annealing at 56°C for 45 seconds, and extension at 72°C for 30 seconds last cycle at 95°C for 30 s, at 56°C for 30 s, and gradual increment from 56 to 95°C at 2°C per minute and lastly for 30 s at 95°C. The cycle threshold (Ct) values obtained were calculated and depicted (Figure 3) (relative quantification) as fold change of IFNA mRNA level in stimulated cells comparison to unstimulated cells as described by Pfaffl [15]. Also the copy number of IFNA transcript/1000 copies of GAPDH in unstimulated PBM cells were calculated as described in our previous study [10]. Statistical significance of differences in the mRNA levels from two species in presence of CpG ODN and PMA were calculated using Student-*t* test.

## 3. Results

### 3.1. Total and Differential Leukocyte Count

It has been evident from Table 1 that TLC count of black buck is less as compared to goat and similarly the DLC count reveal that neutrophil and monocyte counts are more in black buck but lymphocyte count is more in goat.

### 3.2. Sequence Analysis

The recombinant plasmids were characterized by restriction enzyme digestion (for insert release) and nested PCR (140 bp), the nucleotide sequences of IFNA gene fragments were aligned to generate full-length ORF sequences (570 bp) similar to bovine IFNA, which were submitted to NCBI GenBank. Accession numbers were obtained for black buck (*Antelope cervicapra*: **FJ959075**) and goat (*Capra hircus*: **FJ959074**) complete coding sequences. Both IFNA cDNA sequences and translated to amino acid sequences of black buck and goat were aligned using EditSeq (DNA star), with reported sequences from six different species: cattle (Bos taurus; E00135.1), horse (Equus caballus; M14542.1), giant panda (Ailuropoda melanoleuca; DQ392970.1), pig (Sus scrofa; DQ872656.1), and partial sequence of sheep (Ovis aries; AY802986.1) water buffalo (Bubalus bubalis; AY323972.1) using Clustal (W) method of MegAlign (DNA star).

Cross species alignment revealed that black buck IFNA cDNA is 94.7% similarity with goat IFNA, 92.1% with cattle, 94.4% with buffalo, 94.6% with sheep, 82.6% with porcine, and 80.7% with equine and similarly goat IFNA 93.0% with cattle, 95.0% with buffalo, 95.6% with sheep, 82.6% with porcine, and 80.0% with equine IFNA, respectively. Like wise the amino acid similarity of black buck IFNA is 89.5% with goat IFNA, 86.3% with cattle, 88.6% with buffalo, 89.2% with sheep, 68.7% with porcine, and 63.8% with equine and similarly goat IFNA 87.9% with cattle, 90.4% with buffalo, 91.6% with sheep, 70.9% with porcine, and 63.8% with equine, respectively. 

Phylogenetic tree based on evolutionary distances was constructed (Figure 2) from both nucleotide and amino acid sequences using Mega 4.1 [13]. It was found that at both of the levels small and large ruminants have formed separate clusters among ruminants, goat and sheep evolved from common ancestors, similarly cattle and buffalo also evolved from common ancestors, however black buck evolved as being an antelope intermediate between these ruminants suggesting its early divergence during evolution from monogastrics.

### 3.3. IFN Alpha mRNA Expression

Higher copies (1380) of IFNA transcript/1000 copies of GAPDH ware observed in unstimulated goat PBM cells as compared to those in black buck (870). Relative quantification of IFNA mRNA in PBM cells revealed trivial alterations upon culture in the presence of TLR9 agonist like CpG ODN C, and PMA in both the species. The expression (fold increase ± standard deviation) is depicted in Figure 3. On comparing the response of black buck and goat PBM cells towards common agonists, CpG ODN produced more expression of IFNA in black buck (0.99 ± 0.17) as compared to goat (0.79 ± 0.34) whereas PMA produced similar expression in goat (1.05 ± 0.35) and black buck (0.97 ± 0.07). Also the mRNA levels from two species in presence of CpG ODN and PMA were found to be insignificant statistically.

## 4. Discussion

Innate immunity is the first line of defense against the invading pathogen in mammalian vertebrate immune system. The nature and extent of immune status will directly depend on the immune cells. Very little is known about the immune system of wild antelopes. A stronger nonspecific immune response in the wild ostriches (higher phagocytosis and lysozymes) and a stronger specific immune response in the domesticated ostriches have been reported [16]. Higher innate resistance to hemorrhagic disease in white-tailed deer in comparison to domestic species has also been reported [17]. In the present investigation, wild antelope black buck was found to possess higher nonspecific immunity cells like neutrophil and monocyte counts and lower specific immunity cells lymphocyte counts as compared to goat, resulting total leukocyte counts were nearly 1/3rd of the values that of goat. This pattern of TLC and DLC counts is similar to that reported for spotted deer and barking deer [18]. However, raise in interferon-alpha expression in black buck PMB cells was also accompanied by response to CpG ODN stimulation as compared to goat cells. 

IFNA was first identified as an antiviral agent during studies on virus interference by Isaacs and Lindenmann [19]. The sequencing of black buck and goat IFN cDNA was essential to design quantitative real-time PCR for expression analysis, therefore we have cloned and characterized IFNA cDNA of these two species. So far IFNA genes have been cloned from a variety of mammalian species, including human [20], mouse [21] and several avian species such as duck [22], chicken [23], and turkey [24], also Teleost fish [25], and Egyptian Rousette bat (Rousettus aegyptiacus) [26], however only little information is available as far as ruminants are concerned.

**Figure 1:**
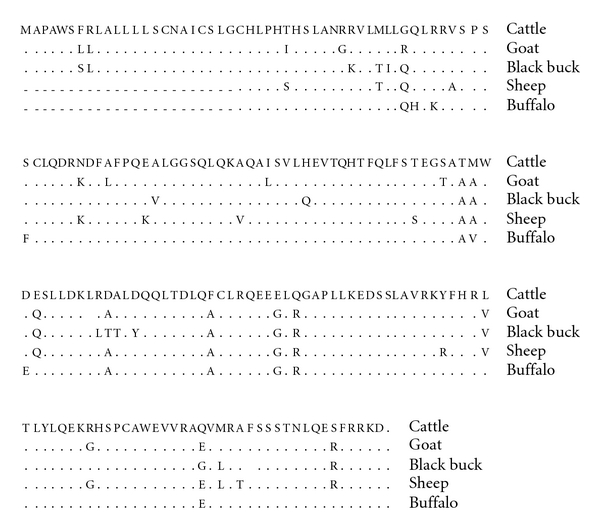
Alignment of predicted amino acid sequence of black buck and goat IFNA with different ruminant species. Identical sequence is indicated by a dot and differences by the corresponding one-letter symbol of the amino acid.

 IFNA gene in black buck and goat was found to have ORF length of 570 bp which is alike to Bos taurus interferon, alpha (NM_174085), and leukocyte (IFN1*α*). Predicted amino acid sequences consist of 189 amino acids (Figure 1), having approximate molecular weight of 21.275 and 21.277 kDa, respectively. First N-terminal 1–23 amino acids are considered to be the secretary signal sequence containing a core of approximately 12 hydrophobic residues, four-amino acid sequence spanning the signal peptidase cleavage site of all class I IFN-*α* proteins, [SLG/C], is also found in black buck as well as goat interferon alpha. Mature mammalian IFN-*α* protein would contain four conserved cysteine residues at positions 1, 29, 99, and 139 amino acids, respectively, [27] present at identical positions in each of the goat and black buck IFNA. Cys29/Cysl39 disulphide bond of the IFNA is considered to be crucial for its antiviral activity. Five conserved proline residues of matured IFNA protein at positions 4, 26, 39, 110, and 139 [27] matched to 4, 26, 39, 110, and 138 positions of IFNA in these two species, inturn revealed that matured peptide consisting of five putative alpha helices which are present in human and porcine IFNA as well indicated A-E [28]. PROSITE analysis revealed that C- terminal amino acids like 146–164 is found to be the IFN alpha, beta, and delta family signature sequence which is also present in goat and black buck IFNA suggesting its functional conservancy in immune system. A consistent stop codon immediately following the terminal lysine (K) codon present in the IFNA of giant panda, sheep, buffalo, and bovine was also found conserved in goat as well as black buck, putative N-glycosylation site [N-X-S/T] which exists commonly in human, feline, and mouse IFNA [21] was not found in any of the two species. Instead it has two potential cAMP- and cGMP-dependent protein kinase phosphorylation sites at amino acid site 45–48, RRVS, and at amino acid site 157–160, KRHS, one Casein kinase II phosphorylation site at amino acid site 103–106, SLLD, was found where S or T is the potent phosphorylation site. Amino acid sequences 153–158 are well conserved among all the species suggesting its common functional role in all the species.

**Figure 2:**
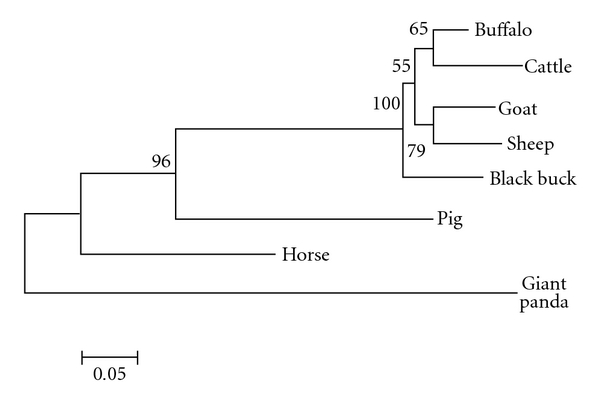
Phylogenetic relationship of the IFNA nucleotide sequences from different species using Mega version 4.1 following the alignment of the ORF sequences using Clustal W and neighbor-joining method (nucleotide p distance). Numbers outside the branches indicate the bootstrap values obtained using 1,000 replicates, and values above 50% are shown. Scale bar at the bottom measures the nucleotide distance.

Based on the nucleic acid sequences of IFNA full ORF region, phylogenetic tree was drawn by Mega 4.1 [13] considering 1,000 bootstrap values (Figure 2). It was found that ruminants and monogastrics like horse, pig, and giant panda form different cluster according to their closer evolutionary relationship. Among ruminants, cattle and buffalo, goat and sheep might have evolved from a common ancestor; black buck positioned in between them diverged early from the bovid ancestors. Selection pressure, as determined by codon-based *Z* test using the Nei Gojobori method, revealed that at 5% level of significance, *dN* is substantially greater than *dS*. Thus, IFNA might have evolved by positive selection (*dN* > *dS*) among these species.

**Figure 3:**
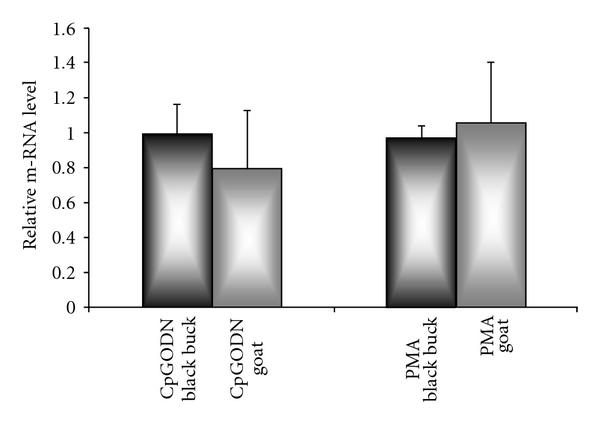
Comparative mRNA expression level of IFNA in PBM cells of black buck and goat stimulated with CpG ODN type-C and PMA (phorbol myristic acetate). Data shown for each biological sample is a (Mean ± SD) of two independent experiments, each runs in triplicates.

Real time quantitative RT-PCR analysis suggested that cycle threshold (Ct) values ranged from 17.23 to 24.66. Melt-curve analysis of IFNA demonstrated two sharp peaks indicating some nucleotide variation between these species. For the first time, marked expression of IFNA transcript in PBM cells was observed in these species. Upon, PMA stimulation produced similar expression of IFNA transcript in goat compared to black buck, however TLR9 agonist CpG ODN produced raise in mRNA expression in black buck compared to goat (Figure 3). Similar findings have been noticed [29] using intestinal protozoa *E. hystolytica* DNA (rich in CpG motifs) is recognized by TLR9 known to increase the IFNA level. Likewise, in felines, it was observed that PMA was known to produce IFNA transcript [30]. 

In response to CpG ODN, level of IFNA transcript was found to be raised in black buck more than in goat PBM cells, these findings are suggestive of stronger innate immune response in wild compared to domestic. Suggesting that the TLR9 primes the innate host immune system against the pathogen derived nucleic acids which are rich in CpG motifs. However the mRNA levels in presence of CpG ODN and PMA were found to be statistically insignificant in these ruminants, suggesting a deeper investigation of interrelationships of a broader range of innate immune cells with cells of adaptive immunity, as well as of expression levels of more than just one proinflammatory cytokine and chemokines would be desirable further.

## Figures and Tables

**Table 1:** Total and differential leukocyte counts in black buck and goat.

	Black buck	Goat
	(Mean ± SD; *n* = 5)	(Mean ± SD; *n* = 5)
TLC/*μ*L	3420 ± 1273	8850 ± 982

DLC in %		

Neutrophil	51.4 ± 6	35.25 ± 6.55
Lymphocyte	44 ± 4	60 ± 7.07
Monocyte	5.4 ± 0.9	3.5 ± 0.96
Eosinophil	00	3.25 ± 1
Basophil	0.6 ± 0.49 (<1)	0
